# Perforations in Duodenal Ulcer

**Published:** 1912-06

**Authors:** Edgar R. McGuire

**Affiliations:** Surgeon Buffalo General Hospital, Adjunct Professor Surgeon University of Buffalo


### Perforations in Duodenal Ulcer.

EDGAR R. Mc GUIRE
Surgeon Buffalo General Hospital,
Adjunct Professor Surgeon University of Buffalo.

IN 1817 appears the first mention of duodenal ulcer in medical literature. It is a case report from Dr. Travers, of a male 35 years old, taken suddenly with excruciating pain in the upper abdomen. For a time the patient was in profound shock; the pain later was referred to the lower abdomen. He died in the bath tub 13 hours following the first onset of pain. Autopsy showed perforation of the duodenum. (Moynihan).

Not until 1894 did Mr. H. B. Dean report the first successful case treated by surgical means. Almost a century has elapsed since Mr. Travers' report, and we are today none too familiar with the diagnosis of this lesion. Within recent times we have been so engaged with the appendix, we have almost lost sight of several other conditions in the abdomen producing acute peritonitis. So prevalent is this opinion that of the total number of operations for perforation of the duodenum, in fully 25 per cent. of them appendectomy was first performed under the errors that the appendix was the cause of the trouble. In my own series of ten cases, this mistake has occurred in two instances. That duodenal perforation is by no means a rare condition, I have but to mention my personal experience of having operated four cases within a week's time.

A discussion on ulceration of the duodenum occurring in extensive burns, forms a part of every treatise on this subject, but the explanation of its causation is most difficult. Similar ulceration occurs in certain cases of Bright's Disease, but these are quite as likely to occur in any other part of the intestine. In general miliary tuberculosis as well as in some instances of peritoneal tuberculosis, ulceration of the duodenum is occasionally seen. These areas are much more frequent in the lower portion of the ileum than any other part of the intestinal tract. Mr. Moynihan, however, has succeeded in collecting 34 cases of tubercular ulcer of the duodenum.

In autopsies of children dying from melena neonatorum, ulceration of the duodenum is not infrequently present.

While these rare conditions must be borne in mind, the chronic ulcer of the duodenum interests us more especially because here perforation is likely to occur. The literature of a few years ago teemed with references to gastric ulcers, occuring chiefly in the pyloric end of the stomach. More careful work showed that these ulcers on the pyloric end were just beyond the pyloric ring, and should strictly be classed as duodenal ulcers rather than gastric. In the majority of instances the ulcer is located so close to the pyplorus, that the name pyloric ulcer might be more expressive of the true condition.

The etiology of these ulcers is obscure. Whatever the cause may be, it seems to be systemic rather than local, because of the frequency with which we find ulcers occuring simultaneously in both the duodenum and the stomach. There are many cases on record where perforation of more than one ulcer has occurred at the same time. The whole subject of the etiology is so wrapped in obscurity there is nothing definite left. The general belief is that they are due to some peculiar toxic action, while again it is thought they are due to some reflex cause such as the appendix. The frequency with which we find a diseased appendix in operation for duodenal ulcer is certainly very suggestive.

Recently Dr. Jacobson, of Syracuse, has discussed the subject of hemorrhage from the stomach and intestine, due to some toxic action. He believes such hemorrhage may come from a general toxemia, when no definite lesion is found in the intestinal canal.

In the developing embryo, two facts stand out of particular importance. First, the nerve supply of the whole intestinal tract with the exception of the upper stomach is such that we have no voluntary control. Consequently, through reflex stimulation, disease anywhere along intestinal tract may produce stomach symptoms, thus making differential diagnosis difficult. Second, in the early stages the intestinal canal is almost a straight tube, later with the development of the caecum the first part of the large bowel moves around to the right side, so that the transverse colon covers the duodenum. This makes the end of the duodenum a pivot point and in the vertical position subject to more or less obstruction. The possibility of this being a causative factor in producing ulcer through back pressure of bile and pancreatic juice must not be overlooked.

Let me quote certain facts from a paper by Dr. E. A. Codman, Boston, Mass. In 3,000 autopsies at the Mass. General Hospital, an open duodenal ulcer was found once in every one hundred cases. Duodenal ulcers were found twice as frequently as gastric ulcers. Duodenal perforations occur 1-20th, to 1-40th, as frequently as acute appendicitis. In Mayo's statistics three-fourths of the cases were males.

The diagnosis of chronic duodenal ulcer is difficult, but far from impossible. Many today believe the diagnosis between chronic appendix, gall-bladder and duodenal ulcer is nigh impossible, and it is only necessary to make a surgical diagnosis, leaving final decision to exploration. When a case is explored by a capable man after a reasonable effort at diagnosis, it is a perfectly justifiable procedure; but, in many instances it is very bad practice because it breeds carelessness in diagnosis, and exposes many innocent medical cases to unnecessary surgical intervention. In the past the frequency with which gastroenterostomy has been done where appendectomy was indicated and vice versa, should teach us to devote more time to previous study in order to prevent similar mistakes in the future. Furthermore, I know of no class of cases where more definite information can be gained by careful study of symptoms alone. I have recently gone over the histories of my cases of perforation, and find they are most typical. Previous to perforation these were all cases of chronic indurated ulcer, and the diagnosis should have been made at that time rather than to wait for perforation to occur. While it is true that embryologically the development of the pylorus and upper intestinal tract is similar, and therefore any disease along the small intestines, gall-bladder, etc., may produce symptoms referable to the pylorus, yet I am satisfied that accurate, painstaking histories of these patients will often reveal the correct diagnosis. Let me quote here from the history of one of my recent cases of perforaion as follows:

"I have always been well up to my present illness, save for some slight stomach trouble. It was never sufficiently serious for me to consult a doctor until three months ago. About ten years ago I noticed that I would have to get up at night because of stomach distress. I have always had my meals at regular hours, 5, 12 and 6 o'clock. The pain has never been severe, but was more a sense of bloating. I found that taking a glass of milk would relieve me, so I frequently had it at hand on going to bed to relieve me during the night. The pain would come on about 11 o'clock. About a year ago I noticed I would have some distress about 4 o'clock in the afternoon, and quite lately I have had a little trouble about 10 in the morning. These attacks of distress were always relieved by taking food, so I got in the habit of carrying something in my pocket to relieve me when the distress would come on. For three or four days before this last attack I was having pain in my left shoulder."

Perforation took place in this patient one morning at 10 o'clock.

It is rather surprising how seldom vomiting occurs. In fact, this is rather a point in differential diagnosis, because in diseases of the appendix and gall-bladder patients frequently experience complete relief following vomiting. While vomiting does occur in cases of duodenal ulcer, it is usually in those late cases with contraction. Hematemesis may be present in either a gastric or duodenal ulcer. On the whole, vomiting is rare in duodenal ulcer, but frequent in the other.

Blood in the stools is of importance. Not every case of duodenal ulcer has even occult blood, but thorough search over a long period will usually show it to be present. Many times it will be learned from the history that the stools were black and tarry at some previous period. Each patient should be definitely questioned in this particular, because the history of the passage of any quantity of old blood from the bowel is very suggestive.

Hyperacidity, or sour stomach is always a constant complaint. Mr. Moynihan goes so far as to state that all cases of untraceable hyperacidity are cases of duodenal ulcer. This seems somewhat extreme, as we know hyperacidity may be reflex from other causes, as a diseased appendix or gall-bladder. It is high time, however, we discarded the term hyperchlorhydria to mean a distinct disease, or while many causes may exist or a temporary hyperacidity (all I think at times have experienced it) yet intractable hyperacidity means definite pathology somewhere. This pathology will usually be found in diseased appendix, gall-bladder, pancreas or duodenal ulcer. The swallowing of a loaded thread is of some importance. If there be found a blood stain above that of the bile, it is suggestive of an ulcer at that point. The distance between the two stains would represent the distance between the opening in the bile duct and the ulcer. In cases of active bleeding an accurate location of nearly the exact point could thus be made.

The statement is frequently made that patients have gall stones for years without symptoms. The same is occasionally heard regarding ulcer of the duodenum. These two statements I believe to be incorrect. The patient will frequently state to the careless historian that he or she has never had any stomach symptoms, but careful examination will reveal the old history of indigestion which the patient probably considered of no importance. I am convinced if more time were given to accurate histories of our patients, we would have fewer mistakes in diagnosis. Laboratory aids to diagnosis are very important, but they should be interpreted with great care to avoid error. On the other hand careful cross questioning of the patient, allowing him to express in his own way his symptoms, will frequently lead to positive diagnosis on the history alone. I would not minimize the value of laboratory, on the contrary would emphasize it, but I would appeal for the same painstaking work on the patients own account of his illness.

Again, at times, we reach a conclusion and then try to make our histories correspond with it. This is reversing the correct order and will lead to error. How often after such mistakes we go over the history with the patient and find a perfect picture of the correct diagnosis had we only properly interpreted it. In a recent case diagnosed as gall stones, I passed over lightly the patient's account of pain in the left shoulder. She barely mentioned the fact and as her other symptoms pointed so positively toward gall bladder disease, I omitted to go into that part of the history carefully. Pain in the *right* shoulder is very frequent in diseases of gall-bladder, liver and duodenum because of the connection between the phrenic and the suprascapular. Pain in the left shoulder goes through the sympathetics pneumogastric. This nearly always means an involvement of the pylorus, either with ulcer involving the peritoneum or in gall bladders adherent to this region. I did find gall stones present, but also found the pylorus densely adherent to the omentum due to pancreatitis. The patient was also tender in the middle of the abdomen, and careful study of her case might have led to a correct diagnosis beforehand. As both conditions were surgical, the matter was not of such great importance in this particular case.

Symptoms in many cases are atypical. Patients may have learned that more pain follows their heaviest meal, and may gradually refrain from eating to relieve them from the severe distress coming three or four hours later. Frequently these patients get so they take nothing but fluids, because while relief follows for a time, they suffer later. Again pain may come within an hour or may be delayed several hours after eating. Mr. Moynihan believes the cases where pain is delayed have an ulcer located posteriorly and some distance from the pylorus. In one of my recent cases distress was said to come sometimes one hour and other times four hours after eating. At operation I found a healed ulcer at the pylorus and an active bleeding one, two and a half inches below the pylorus on the posterior wall of the duodenum. The patient was in poor condition, but under nitrous oxide and oxygen, I placed a circular suture around ulcer, completely controlling the bleeding. The hemoglobin rose from 25 per cent. to 56 per cent. in two weeks, and in eight weeks had risen to 85 per cent.

Briefly then when a patient complains of persistent distress from one to two hours after eating, with recurring attacks relieved by food, no vomiting, hyperacidity, a diagnosis of chronic duodenal ulcer can be made with considerable confidence. The matter of recurring attacks is important. In almost every case there is a complete relief of symptoms, but it is only for a time, varying from a month to several years. This periodicity explains many medical cures, and led one surgeon to say he never operated for ulcer until it was cured nine times by medical means.

Perforation of a chronic ulceration of the duodenum is one of the most serious conditions with which the surgeon has to cope. While I am quite aware of the excellent results following early suture in the acute cases, and drainage in the late localized forms, yet when a patient apparently well can be dead in twenty-four hours from the first onset of pain, we are dealing with a most serious disease. There are many classifications given of these cases, but the simplest from a clinical point of view is acute, sub-acute and chronic perforating ulcers. By the acute, I mean those with no attempt at walling off; by the sub-acute, those in which a piece of amentum or lymph temporarily occludes the opening and usually a large sub-diaphragmatic abscess occurs; and by the chronic, those which slowly perforate forming adhesions, and become entirely walled off.

Necessarily the mortality in these cases will vary according to type of peritonitis present. It is very low in the chronic, comparatively low in the sub-acute, but in the acute cases it must be estimated largely by one factor; namely, the length of time elapsing from the time of perforation to that of the operation. Spreading peritonitis from this cause differs in no essential particular from that following rupture of the appendix, where with each succeeding hour the mortality increases. If we could operate our appendices before perforation occurs, our mortality would be practically zero; and so could we but operate the chronic ulcers before perforation occurs, the mortality would be correspondingly low. Are there any premonitory symptoms or signs which would lead us to make a diagnosis of beginning perforation? In probably the majority of instances perforation comes out of a perfectly clear sky, and the patient is almost immediately overwhelmed; but in quite a proportion of my own cases there have been symptoms which, if properly interpreted, might have led to a diagnosis of oncoming disaster. In three cases, for several days before perforation occurred, the patient had more pain and more local tenderness. This was sufficiently marked for them to mention it in their histories. In two of the cases pain in the shoulder occurred two days before perforation. If I interpret these symptoms correctly, they were the results of an involvement of the peritoneal coat of the bowel, and showed that only a thin layer separated the bowel from perforation. In my other cases there were no premonitory symptoms, unless we mention the old history of indigestion. This was present in all my cases. If we studied our cases thoroughly, we would know chronic ulcer to be present in that individual, and when acute pain developed, it would make the diagnosis comparatively easy.

Acute perforation of a chronic duodenal ulcer usually presents a perfectly typical picture. Exceptions may occur, but in the main they follow closely the picture here described. It is something different from any other abdominal catastrophe unless it be perforation of a gastric ulcer. The pain is very sudden and very severe. Patients assume an attitude bent over in bed, with thighs flexed on the body, eyes protruding, facial expression pinched and anxious, and extreme rigidity of abdominal muscles. It is different from any other abdominal lesion with which I am familiar. In peritonitis from an appendix, one finds a rigid abdomen, but not until late does it assume an absolutely board-like feel on both sides. Here also the abdomen is distended; but, in perforating ulcer, however, the abdomen is flat and almost immediately board-like rigidity is present. This is evidently caused by the sudden outpour of duodenal contents into the peritoneal cavity rather than by an infectious process, because sufficient time has not elapsed for infection to produce it. If seen very early, rigidity may be confined to the upper abdomen; if in a few hours, the whole body will be rigid but not distended. Distention comes on later. The pain and rigidity go together; first, in the upper abdomen, then down the right side to the pelvis and later over the whole abdomen.

In this condition I have often been impressed with the difficulty of accurately diagnosing fluid in the peritoneal cavity in cases of perforation. It does seem as though the presence of gas in the peritoneal cavity renders the precussion note resonant, even though fluid be present. In typhoid perforation I have known four or five good men to agree there was no fluid in the peritoneal cavity and at operation find quantities of free intestinal contents.

The leukocytes in my cases varied from twelve to thirty thousand. The count is of some value in indicating intervention, but of very little help in differential diagnosis. In one of my patients I was able to elicit a sign which I have never before seen mentioned in this connection. I was fortunate in seeing this case within two hours of the time of perforation. It was a typical case, and listening with a sethoscope over the duodenum, I was able to hear fluid and gas gurgling through the opening in the bowel. This, of course, might have been conflicted with the same process inside the bowel, but it was distinctly heard by two others beside myself and I believe it to be of value in some cases if seen sufficiently early. Through delay in obtaining the family's consent, operaperforation, and at this time we could hear nothing over the duo-
perforation, and at this time we could hear nohing over the duo-
denum.

Treatment of this condition is in every particular that of the treatment of peritonitis. In the chronic forms usually it will be only necessary to open and drain what is really a localized abscess, as the opening in the duodenum has probably closed as a result of adhesions. In any event it is rather dangerous practice to break through a well defined barrier to the spreading of the infection, even though the opening be still patulous.

In the sub-acute variety we have to deal with a somewhat different condition. Usually we have a rather large sub-diaphragmatic abscess, and in one or two instances I have seen there was very little attempt at walling off. In this type of case I would feel much more secure if the opening in the duodenum were closed, but on the other hand it might be bad practice if any walling off had occurred. Probably a middle ground is the safer one to pursue, feeling at the same time if the opening could be closed without too great a risk it would be preferable. Anterior drainage of these cases is often inefficient; posterior drainage through the flank being better in some instances. On the other hand less drainage will be needed if the opening in the duodenum be closed.

In the acute variety we are dealing with exactly the same condition as found in spreading peritonitis due to any other cause. Here I believe the method of treatment is fairly well established: First, to *quickly* close the opening whether it be from appendix, gall bladder or ulcer; and second, to establish adequate drainage.

In the treatment of these cases time is a very important factor. The sutures are previously threaded, and the operator and assistants ready for work before the anesthetic is started. I have used as a rule either nitrous oxide or ethyl chloride anaesthesia, a little ether at times being necessary. In this way the patient is not usually under an anaesthesia over fifteen minutes. Fowler position is used as soon as the diagnosis is suspected. In most of my cases I found patients sitting up in bed on my arrival. In spreading peritonitis it is quite as important to establish the sitting position before operation as following it. The matter of irrigation is still a very greatly disputed question. I, personally, never irrigate any of these cases; in fact, I seldom irrigate any wound.

Regarding the matter of closing the perforation in these cases, there is quite a diversity of opinion. At times I have experienced considerable difficulyt in delivering the duodenum into the wound, but after dividing the peritoneal fold on the upper surface of the duodenum, this can usually be accomplished in exactly the same way as is done in doing a Finney gastroduodenostomy. I have usually had trouble with the purse string suture because it was placed in friable tissue and would pull out. Consequently of late I have made an oval incision of the ulcer, closing the wound in the reverse direction after the manner of the ordinary pyloroplasty. While this may be of considerable benefit in the local treatment of the ulcer, yet the greatest benefit I think lies in securing more accurate closure. I have never known one of these cases to leak, although my experience has been limited. Dr. Elliott has shown in animals, no matter how extensive the excision of the wall of the duodenum, it is almost impossible to have permanent contraction occur. If this be true, it would seem to settle the question of gastro-enterostomy. In these cases if an anastamosis is to be considered, the operation of Finney is much preferable to the gastrojejunostomy. In the main, however, these cases are sufficiently serious to prevent doing more than accurate closure of the opening. If contraction should occur, the time for an anastamosis is when the patient has recovered from his peritoneal infection, rather than give any added risk at this time. I drain these cases with a stab wound in the pelvis, closing the primary opening. In one or two instances I have drained the upper wound where I have been afraid of leakage. All cases of spreading peritonitis and treated by the Fowler-Murphy method.

Mortality in acute cases varies with different operators, dependent almost entirely upon the length of time between the first onset of pain and the operation. Mr. Moynihan reports 11 cases with 3 deaths. Mitchell of Belfast has had 19 cases with 18 recoveries; he has had the unusual experience of seeing this large number operated early. The report of deaths has usually occurred in cases extending over 15 hours after perforation. In view of this fact, how important early diagnosis becomes.

In my 10 cases of perforation, there have been 7 acute cases with 1 death; 3 sub-acute with 1 death. I believe the death in the acute case was due to patient refusing operation until 18 hours after perforation. In the sub-acute case, there was some walling off as I saw the case 8 days after perforation. I simply drained this immense cavity containing literally quarts of fluid without attempting closure of the perforation. The patient lived about a week, finally dying with rales over both bases and generally septic. Autopsy was refused, but the old incision was opened and perforation was found about two inches below the pylorus on the posterior wall of the duodenum.

Case reports are here omitted, but in reviewing same I find there have been seven (7) acute cases with one death. They were all males with an average age of forty-two (42) years. The average length of time between perforation and operation was seventeen (17) hours. In the one case failing to recover, operation was performed eighteen (18) hours after perforation. In the sub-acute variety there were three (3) cases with one death. This occurred one week after operation from general sepsis. The chronic cases have been much more frequent but they are not included in this report, as operation is usually nothing more than draining a well walled off abscess.

In closing, I would urge particularly: first, accurate histories taken of our stomach cases, as a diagnosis can frequently be made from this alone; second, particular care in chronic ulcer when superficial tenderness and pain in either shoulder be present; third, operation in perforated ulcers at the earliest opportunity; fourth, excision of the ulcer with suture in the reverse direction; fifth, gastroenterostomy is contraindicated in perforating ulcers; after excision contraction is almost impossible.

